# A Hybrid Deep Learning Model for Recognizing Actions of Distracted Drivers

**DOI:** 10.3390/s21217424

**Published:** 2021-11-08

**Authors:** Shuang-Jian Jiao, Lin-Yao Liu, Qian Liu

**Affiliations:** Department of Civil Engineering, College of Engineering, Ocean University of China, Qingdao 266100, China; jsj6039@ouc.edu.cn (S.-J.J.); lly6409@stu.ouc.edu.cn (L.-Y.L.)

**Keywords:** driver distraction, OpenPose, LSTM, keyframe sequences, action recognition, nested cross-validation

## Abstract

With the rapid spreading of in-vehicle information systems such as smartphones, navigation systems, and radios, the number of traffic accidents caused by driver distractions shows an increasing trend. Timely identification and warning are deemed to be crucial for distracted driving and the establishment of driver assistance systems is of great value. However, almost all research on the recognition of the driver’s distracted actions using computer vision methods neglected the importance of temporal information for action recognition. This paper proposes a hybrid deep learning model for recognizing the actions of distracted drivers. Specifically, we used OpenPose to obtain skeleton information of the human body and then constructed the vector angle and modulus ratio of the human body structure as features to describe the driver’s actions, thereby realizing the fusion of deep network features and artificial features, which improve the information density of spatial features. The K-means clustering algorithm was used to preselect the original frames, and the method of inter-frame comparison was used to obtain the final keyframe sequence by comparing the Euclidean distance between manually constructed vectors representing frames and the vector representing the cluster center. Finally, we constructed a two-layer long short-term memory neural network to obtain more effective spatiotemporal features, and one softmax layer to identify the distracted driver’s action. The experimental results based on the collected dataset prove the effectiveness of this framework, and it can provide a theoretical basis for the establishment of vehicle distraction warning systems.

## 1. Introduction

According to data published by the World Health Organization (WHO), approximately 1.2 million people die in traffic accidents worldwide every year [1]. According to the National Highway Traffic Safety Administration (NHTSA), approximately 20% of traffic accidents and 80% of almost impending traffic accidents are caused by driver distraction, which emerges as a key factor in serious and fatal accidents [2]. In 2018 alone, driver distraction claimed the lives of 2841 people in the USA [3]. Therefore, investigating the cause of distracted driving and reducing the number of distraction-affected traffic accidents remains an imperative issue.

According to related research [4], there are two main reasons for driver distraction: (i) internal reasons: fatigue driving, drunk driving, and drug driving, that is, the mental states of the driver are not suitable for driving. Methods that focus on detecting driver distraction due to internal reasons are mainly divided into physiological parameter-based methods [5,6] and naturalistic driving data-based methods [7,8]; (ii) external reasons: the driver has external interference, such as calling, texting, and talking with passengers, and other secondary tasks that interfere with the driver driving in the proper mental condition. Computer vision methods are used to identify driver distraction caused by external reasons, which have two advantages that can put them into practical application. First, compared to the physiological parameter-based methods, it is a non-intrusive technique of obtaining data, which can ensure that the drivers are not affected by the measuring instruments. Second, compared to the naturalistic driving data-based methods, it can warn the drivers after they have performed distracted actions, instead of warning them after the vehicle’s behavior has already become abnormal.

Against this contextual backdrop, we focus on driver distraction caused by external causes, that is, we choose to use computer vision methods to detect actions of distracted drivers. Driver action recognition (DAR) is a branch of human action recognition (HAR). In the HAR field, the two major aspects in developing deep networks for action recognition are the convolution process and temporal modeling [9,10]. Moreover, dealing with the temporal dimension is a challenging issue. The current mainstream solutions include three major categories: two-stream convolutional networks [11], three-dimensional convolutional networks (3D-ConvNets) [12], and fusion of convolution neural networks and long short-term memory [13] (CNN-LSTM). Table 1 gives a brief introduction of the architectures with advantages and disadvantages. Because CNN-LSTM architecture has high accuracy and fast speed, it was selected as the basic architecture of this research. However, simply completing the architecture selection is not enough, because HAR systems are not automatically useful under DAR constraints. The limited in-vehicle space where the actions are executed and the parallel execution of different in-vehicle actions with driving tasks drastically challenge the HAR techniques [14]. Therefore, the problem that needs to be solved urgently at this stage is how to extract efficient temporal and spatial features of the driver’s actions, to effectively identify the different actions of the drivers.

In this paper, a hybrid deep learning model is proposed to recognize the actions of distracted drivers. This model uses the OpenPose skeleton extraction algorithm, essentially a CNN model, to obtain the skeleton information of the human body (including bone maps and the joint point position information) by processing every frame captured by monitoring. Then, the action description features (ADFs) are constructed by using the joint point. Based on this, the ADF vectors are composed of the vector angles and the modulus ratio of each frame, which are used as the input of the K-means clustering algorithm to preselect the original frames. Then the keyframe sequences are obtained by using inter-frame comparison (IFC). Finally, the ADF vectors representing the keyframe sequences are fed into the LSTM, which then outputs the recognition results. The model we proposed improves recognition accuracy through a combination of the following three processes: (i) combines OpenPose and LSTM as the basic architecture guarantees the extraction of spatiotemporal features; (ii) constructs ADFs, which realizes the fusion of deep network features and artificial features. The proposed ADFs improve the information density of spatial features and, to a certain extent, eliminate the influence of individual differences and changes in shooting distance; (iii) uses K-means clustering algorithm and IFC to extract keyframe sequences, which can reduce the interference of similarity of actions of distracted drivers and action speed on recognition.

There are three major contributions of this paper.

- We propose a novel model, which avoids the use of complex devices (i.e., wearable sensors [15,16] and depth cameras [14]) and only needs 2D cameras in vehicles.
- A highly efficient method is introduced to handcraft an effective spatial feature based on the joint points (i.e., deep neural features) derived from OpenPose.
- The introduction of temporal features extracted by the K-means clustering algorithm, IFC, and LSTM networks, which makes up for the current deficiencies in the DAR field.

The outline of this paper is as follows. Related works and the current state of researches are reviewed in Section 2. Section 3 elaborates on the data collection process. In Section 4, our model with four modules is described in detail. The experimental results and analysis are presented in Section 5. Additionally, this paper will be finalized with conclusions and a forward-looking emphasis in Section 6.

## 2. Literature Review

The main focus of our research is to extract efficient spatiotemporal features from driver action sequences so as to improve the accuracy and robustness of driver distracted action recognition. Therefore, we summarize and review from three aspects: the application of the computer vision method in the DAR field, the spatiotemporal features acquisition based on skeleton data, and the current status of keyframes extraction.

Many researchers have applied computer vision methods to the field of DAR. Li et al. [17] situate and detect the driver’s right ear and right hand using You Only Look Once (YOLO) and take the coordinates of regions of interest (ROIs) as input, and a multi-layer perceptron is designed to infer the driver’s status from the ROIs. Huang et al. [18] present a hybrid CNN framework (HCF) combining Xception, Inception V3, and ResNet50 to detect the actions of distracted drivers, which can improve the accuracy of the driving activity detection system. Baheti et al. [19] propose a new architecture named mobileVGG based on depth-wise separable convolutions for detecting and classifying the driver distraction, which greatly reduces the parameters compared with other CNN models. Mase et al. [20] introduce a novel method using CNNs and stacked bidirectional long short-term memory networks (BiLSTM) to capture the spectral-spatial features of the images, where BiLSTM is used to handle the sequence of filtered channels, that is, the output of CNNs (i.e., 8 ∗ 8 feature maps with 2048 channels). Omerustaoglu et al. [21] integrate the prediction results of the vision-based CNN and the sensor-based LSTM model into the final model to obtain the driver’s distracted motion detection results, which improves the accuracy and generalization ability of the system. For the chaotic driving scene, Jegham et al. [14] use an RGB-Depth camera to capture RGB images and propose a novel soft spatial attention-based network. It can be summarized that, in the field of DAR, most researchers only focus on the combination and improvement of the CNN model, striving to improve the accuracy or speed of the static detection model, but ignore the importance of temporal information. Although, as the research further develops, the architecture of CNN-LSTM has begun to be used by some researchers to recognize driver’s distracted actions, it still needs additional equipment such as sensors and depth cameras.

With the rapid development of pose estimation techniques, action recognition based on skeleton data is a research hotspot. Skeleton data is the characteristic information of the joint points obtained from the action sequence, including relative track, position, and so on. Wu et al. [22] extract the meaningful temporal features of sub-actions from the three-dimensional skeleton data by a multiscale wavelet transform, which can improve the robustness of action recognition. Zuo et al. [23] propose two new graph convolution methods: the partial-image convolution network and full-image convolution network to learn the part scale spatiotemporal features and full-scale skeleton spatiotemporal features. Then the two features are combined to obtain more effective skeleton features. For improving the performance of action recognition, Ahad et al. [24] regard 3D bone joints as kinematic sensors based on the three-dimensional linear joint position and the angle between the bone segments and propose the linear joint position feature and the angular joint position feature. Ma et al. [25] use the distances and angles between the joint points as spatial features to input to the deep graph convolutional network (DGCN) and LSTM, which can complete action recognition of basketball players. Connecting the lines between the same joints in adjacent frames, Tasnim et al. [26] propose a 3D spatiotemporal image formation technology of skeletal joints by capturing spatial information and temporal changes for action discrimination. Above all, previous studies have processed the acquired skeleton data to different degrees to concentrate the information of the action sequence, which can make the model capture relatively more information for training. The above review also shows that the fusion of heterogeneous features, namely handcrafted and deep neural features, can improve the robustness of action recognition by analyzing action sequences from different aspects of expert views and data-driven model views, respectively.

Many studies have shown that using only a few keyframes instead of a complete sequence of frames can perform action recognition tasks more effectively and summarize the video [27,28,29,30,31,32]. Kim et al. [33] prove that the keyframe extraction enables fast and robust gesture recognition regardless of motion speed. Wang et al. [34] extract an energy feature, combining kinetic energy and potential energy, from 3D video sequences to represent human actions and employ a support vector machine (SVM) to recognize human actions on the EFs of selected keyframes. Tang et al. [35] combine image density clustering and entropy and use keyframes in gesture videos for further feature extraction to improve recognition efficiency. Yasin et al. [36] extract the keyframes that contribute to the action performance from the motion sequence of the 3D frame to eliminate redundant frames and summarize the motion sequence while retaining the original motion semantics. It can be seen that the keyframes should be representative of the video content, diverse to reduce the redundancy, and should be able to cope with the impact of movement speed on recognition.

To summarize, in the field of DAR, most researchers only focus on combining deep learning models to try to extract spatial features with higher information density, ignoring the importance of temporal information. The method proposed in this paper not only introduces a spatial feature extraction method distinguishing from the existing techniques but also extracts the temporal features of the driver’s distracted action. To the best of the authors’ knowledge, the methods of obtaining the spatiotemporal features based on RGB video sequences had yet, to date, to be fully researched. The model embedding feature construction method based on bone information and keyframe sequences technique fills the gaps in the DAR field, which can eliminate the influences of individual differences and movement speed.

## 3. Data Collection

The literature shows that the driver’s distracted actions mainly include eating, drinking, manipulating dashboard controls, watching a smartphone screen, talking on a phone or with passengers, and grooming [37]. Therefore, in this study, the above seven actions are selected as the distracted actions to be recognized. The State Farm Distracted Driver Detection dataset published on Kaggle [38] and the American University in Cairo (AUC) Distracted Driver Dataset [39] are the most frequently used datasets in the related studies. However, they cannot meet our needs because of the following two reasons. First, the images in the dataset extracted from the same video are almost identical to each other. Second, there is no timestamp information or sequence information about the images [21,39,40]. Therefore, we created a new dataset. In order to make our data collection reasonable, the custom dataset is collected by mimicking the State Farm dataset (e.g., the camera perspective, distance, and the scenarios).

**Figure 1:**
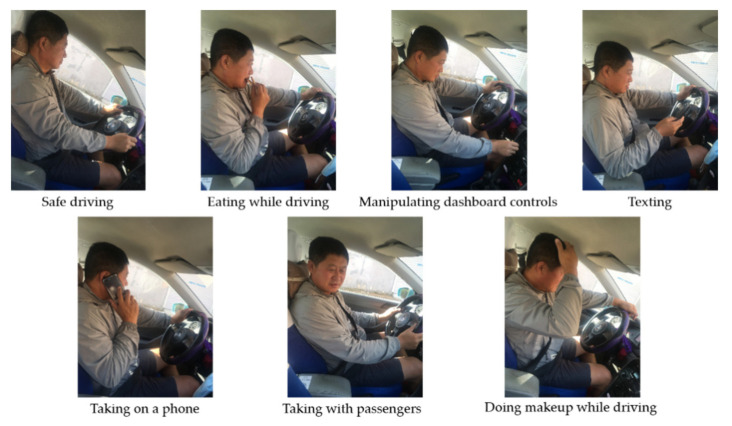
Some sample frames of distracted actions from the custom dataset.

Figure 1 and Table 2 show the examples of the custom dataset, which contains 8 types of actions performed by 5 females and 10 males of various heights and body shapes. While the vehicle was moving, we collected 30 frame-per-second videos, and each video is controlled at 3 s, so that the length of each action sequence is 90 frames. It is worth noting that performers must demonstrate actions C0, C3, C4, C5, and C6 twice at different speeds, and demonstrate actions C1, C2, and C7 three times each, thus forming a custom dataset containing 285 action sequences. That means we collected about 25,650 data points with timestamp information. A mobile phone, which was placed in the upper right corner of the vehicle, was used to collect video sequences. This location was chosen by us because a similar placement was used in the State Farm dataset.

## 4. Methodology

**Figure 2:**
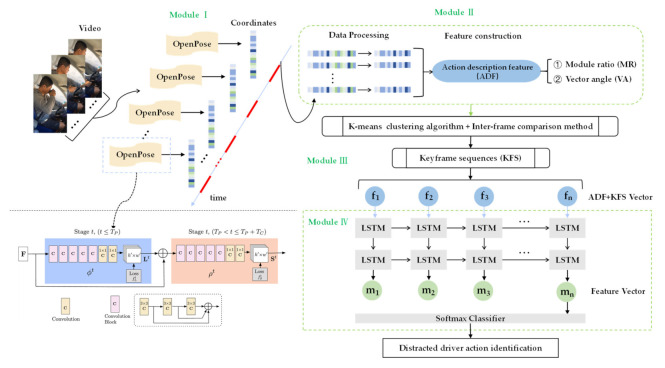
The architecture of our proposed model.

The architecture of our proposed model, as shown in Figure 2, consists of a list of modules: the module of human body poses estimation (Module I), the module of data processing and feature construction (Module II), the module of keyframe sequences extraction (Module III), and the module of action recognition (Module IV). 

### 4.1. Module I

**Figure 3:**
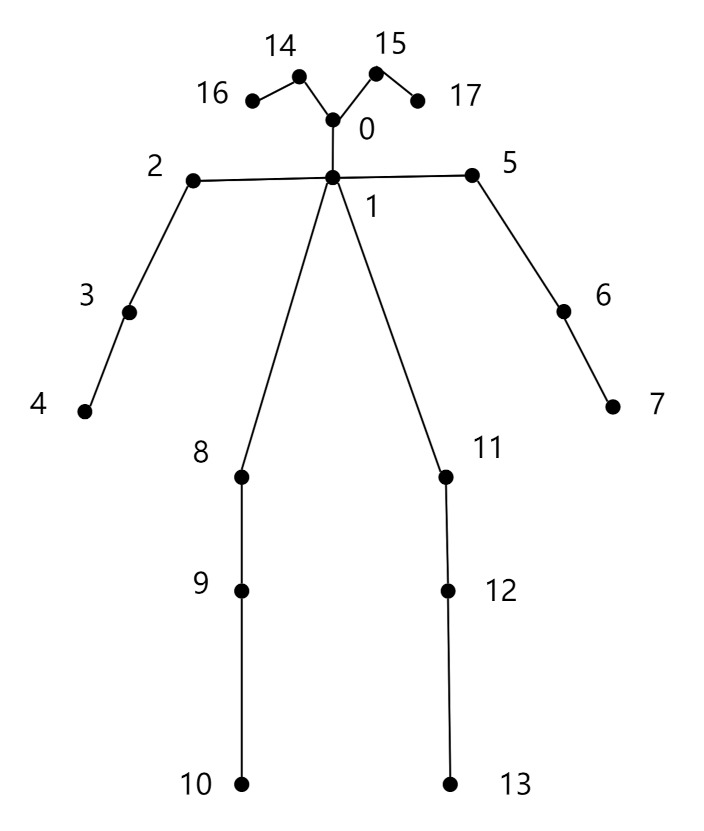
Eighteen joint points of the human body.

We chose the OpenPose algorithm [41], first proposed by the Perceptual-Computing-Lab of Carnegie Mellon University, as a technique of detecting human joint points because of its high accuracy. After several generations of updating and optimization, as shown in the bottom left of Figure 2, the latest OpenPose algorithm reduces the computation amount by half compared with the original structure, but the accuracy almost remains unchanged, which is suitable for obtaining skeleton data. This algorithm was first applied to the COCO key challenges, greatly surpassing the previous results [42]. The reason why we also choose the COCO model is its characteristics of generating 18 joint points to provide a good trade-off between a detailed representation of the human pose and complexity. Table 3 shows the 18 joint points saved in each frame of OpenPose. Figure 3 demonstrates the 18 joint points of the human body. The data of each joint point includes the abscissa value and the ordinate value in the Cartesian coordinate system and the confidence.

**Figure 4:**
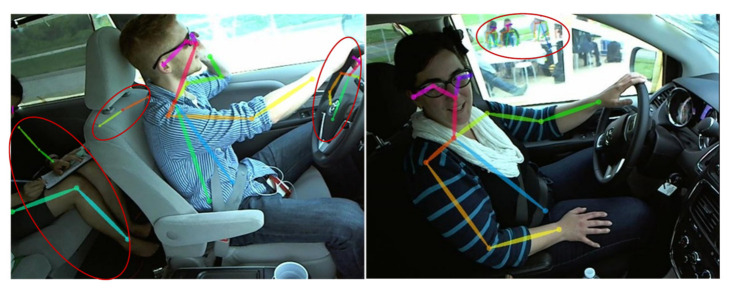
Misidentification cases.

However, applying the OpenPose algorithm may be difficult in the following cases. First, as shown in Figure 4, multiple human skeletons appear in a frame. Second, body occlusion can lead to localization error and false negatives. Therefore, we set up the data processing unit in Module II, which handled the above cases properly.

### 4.2. Module II

This module is mainly divided into two parts, namely data processing and feature construction. First, we process the collected coordinate data of the joint points. Second, in order to improve the information density of spatial features and make our proposed model have better characteristic performance, we construct the vector angle and vector modulus ratio based on the processed joint point coordinates as in the ADFs.

#### 4.2.1. Data Processing

Objects or pedestrians are sometimes mistaken for human skeletons by OpenPose, causing multiple human skeleton information to be stored in the json file. To cope with this phenomenon, we compared a large amount of data and found that the skeleton information with the highest confidence is displayed on the first line of the json file. In other words, the skeleton information is sorted by confidence, which reaches the threshold but has the lowest confidence is arranged at the end of the file. Since the focus of the video is on the driver, it is clear that the driver’s skeleton is the most obvious and the confidence is the highest. Therefore, the first human skeleton information in the file is always retained.

Since the driver’s distracted action only includes the upper body, we deleted the joint point data numbered 9, 10, 12, and 13 in Table 3 and all confidence values to avoid the interference of irrelevant data. Because the shooting angle of the dataset has caused a large loss of the performer’s left ear joint points, as shown in Figure 4, the joint point data numbered 17 were also deleted. Some of the existing methods for dealing with missing joint points are as follows: (i) handle the missing data by model. For example, in Xgboost [43] and Light GBM [44], the model skips the missing values and calculates directly; (ii) a statistical method that replaces the missing value with the mean, median, and plural [45]; (iii) the valuation of missing data using the Kalman filter [46], currently the most reliable method. In our experiment, the missing values can be well supplemented by statistical methods because there are few missing points. In order to make the coordinates of missed joint points in frames be completed, the detailed procedure of the mean-coordinate supplement method (MCSM) we proposed is as follows. We divide the joint points into two categories: (i) fixed joint points, that is, the joint point where the position remains unchanged, as shown in Figure 3 as 1, 8, 11, and (ii) changing joint points, that is, 0, 2, 3, 4, 5, 6, 7, 14, 15, and 16 in Figure 3. The movements are mainly reflected by these joint points related to the arm and head. The processing methods are as follows:

(i) Fixed joint points: We take the average of all unmissed joint point coordinate data to replace the joint point coordinates of all video frames. The formula is as follows:

(1) $$\left\{\begin{array}{l} x_{j}=\frac{\sum x_{j}^{m}}{M}\\ y_{j}=\frac{\sum y_{j}^{m}}{M} \end{array}\right.$$

where, j represents the fixed joint points, m is the frame without missing j joint point data, M is the number of frames without missing j joint point data, and $x_{j}$ and $y_{j}$ are the coordinate values that replace the abscissa and ordinate of the j joint point in each frame.

(ii) Changing joint points: There are three possible scenarios. First, single data is missing. The missing coordinate of the frame is represented by the average value of the data of the K frames before and after it, and the formula is as follows:

(2) $$\left\{\begin{array}{l} x_{j}^{i}=\frac{\sum_{k=1}^{K}x_{j}^{i-k}+x_{j}^{i+k}}{2K}\\ y_{j}^{i}=\frac{\sum_{k=1}^{K}y_{j}^{i-k}+y_{j}^{i+k}}{2K} \end{array}\right.$$

where, j represents the changing joint point, i is the frame with missing j joint point, and $x_{j}^{i}$ and $y_{j}^{i}$ are the coordinate values that replace the abscissa and ordinate of the j joint point in the frame i. Experiments on non-missing joint points show that the average value of the two frames before and after the data is optimal, that is, K=2.

Second, consecutive data is missing. Formula (3) shows the supplementary method of i to i+n−1 frames which continuously misses n frames. Third, if the joint point data for the first frame of the action sequences are lost, the mean of all unmissed joint point coordinate data is taken and supplemented to the first frame. From then on, we can continue to process the data using the above two processing methods.

(3) $$\left\{\begin{array}{c} x_{j}^{i}=\frac{x_{j}^{i-1}+x_{j}^{i+n}}{2}\\ x_{j}^{i+1}=\frac{x_{j}^{i}+x_{j}^{i+n}}{2}\\ \ldots \ldots \\ x_{j}^{i+\left(n-1\right)}=\frac{x_{j}^{i+\left(n-2\right)}+x_{j}^{i+n}}{2} \end{array}\right.$$

The data after the above processing is stored in the following format. (i) Each frame of data occupies a separate row, arranged in chronological order, with 2-row indexes, which are the person and the action of the row of data. (ii) Each row contains 28 columns of data, which are the coordinate values of the abscissa and ordinate of the above-mentioned 10 changing points and 3 fixed points in a rectangular coordinate system.

#### 4.2.2. Feature Construction

If the processed 13 joint-points coordinate data are directly used for subsequent operations, the generalization ability of the model is low. Based on the coordinate data of the joint points, we artificially construct the ADFs, that is, the vector angle and the modulus ratio of the human body structure to achieve a more effective feature descriptor for action recognition [47]. Furthermore, through the analysis of the characteristics of the driver’s actions, two auxiliary points, an improvement for specific application scenarios, are creatively proposed to assist in the construction of ADFs. The detailed process is as follows:

**Stage 1.** Acquisition of structure vector. The calculation method is to subtract the coordinates of two joint points in the same frame, the formula is as follows:

(4) $$l_{j1,j2}=(x_{j1},y_{j1})-\left(x_{j2},y_{j2}\right)$$

where, $\left(x_{j1},y_{j1}\right)$ and $\left(x_{j2},y_{j2}\right)$ is the coordinate of the $j_{1}$ and $j_{2}$ joint points, and $l_{j_{1},j_{2}}$ is the structure vector composed by the $j_{1}$ and $j_{2}$ joint points.

**Figure 5:**
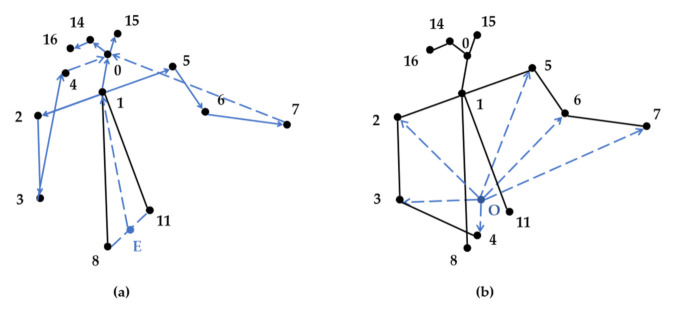
The human body structure vectors (the solid and dashed lines denote human skeleton and non-skeleton respectively, the blue line represents the human body structure vectors) (**a**,**b**).

This paper constructs 19 structure vectors for the subsequent calculation of vector angle and vector modulus ratio, as shown in Figure 5. The innovative point of this paper is the creation of point E (the midpoint of fixed points 8 and 11) and the point O (the center of gravity of the triangle formed by fixed points 1, 8, and 11). The creation of point E is helpful for the subsequent calculation of the modulus ratio, and the creation of point O is helpful for a better description of upper limb movements. Taking the joint point 3 (right elbow) in Figure 5 as an example, the construction of the structure vectors $l_{2,3}$, $l_{3,4}$ and $l_{o,3}$ can well describe the movements related to the right elbow.

**Stage 2.** Acquisition of vector angle. The angle value between the vectors is calculated using the law of cosines. The calculation formula of the vector angle is:

(5) $$\theta_{\alpha }=\langle l_{j1,j0},l_{j1,j2}\rangle =\arccos\frac{l_{j_{1},j_{0}}\cdot l_{j_{1},j_{2}}}{|l_{j1,j0}||l_{j1,j2}|},\alpha \in \left\{0,\cdots ,13\right\}$$

where, $l_{j_{1},j_{0}}\cdot l_{j_{1},j_{2}}=\left(x_{j_{0}}-x_{j_{1}}\right)\left(x_{j_{2}}-x_{j_{1}}\right)+\left(y_{j_{0}}-y_{j_{1}}\right)\left(y_{j_{2}}-y_{j_{1}}\right)$, $\left|l_{j_{1},j_{0}}\right|=\sqrt{{\left(x_{j_{0}}-x_{j_{1}}\right)}^{2}+{\left(y_{j_{0}}-y_{j_{1}}\right)}^{2}}$ and $\theta_{\alpha }$ is the angle between vectors $l_{j_{1},j_{0}}$ and $l_{j_{1},j_{2}}$.

**Figure 6:**
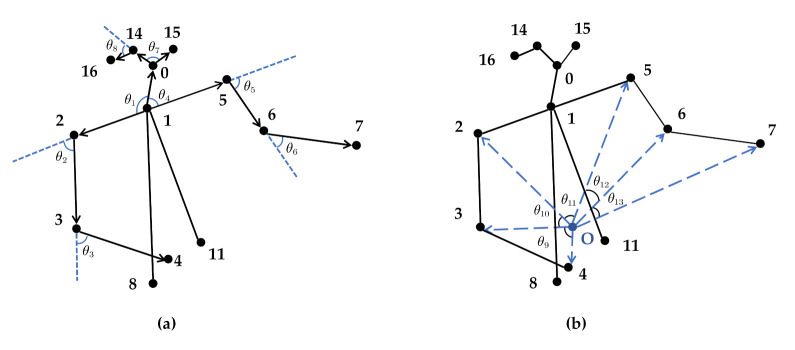
Vector angle of human body structure (**a**,**b**).

This paper constructs 13 vector angles, as shown in Figure 6. Additionally, taking the number 3 (right elbow) joint point as an example, $\theta_{3}$ is the angle between the upper arm and the forearm in Figure 6a, which can be used to measure the swing angle of the forearm relative to the upper arm. $\theta_{9}$ and $\theta_{10}$ in Figure 6b respectively represent the angular relationship of the right elbow joint with respect to the right shoulder, right wrist, and point O. The unique position of the joint point can be determined by the above three vector angles.

**Stage 3.** Acquisition of vector modulus ratio. In order to avoid large errors in the recognition of driver action due to individual differences, this paper does not use the absolute distance between the joints but chooses the relative distance, that is, the vector modulus ratio. In our paper, a total of eight vector modulus ratios have been constructed, as shown in Table 4. Equation (6) gives a calculation example of the vector modulus ratios $r_{1}$ and $r_{7}$:

(6) $$r_{1}=r_{O,2}=\frac{|l_{O,2}|}{|l_{E,1}|},r_{7}=r_{4,0}=\frac{|l_{4,0}|}{|l_{E,1}|}$$

The distance between the midpoint E and the joint point 1 can be almost constant during driving, and it can well reflect the body shape of different humans. Therefore $l_{E,1}$ is selected as the base vector to calculate the vector modulus ratio, which can eliminate the individual differences between different drivers.

In this paper, 13 vector angles and 8 vector modulus ratios of human body structure are constructed as the features of the driver’s actions, totaling 21 ADFs.

### 4.3. Module III

In this section, we propose a module based on the K-means clustering algorithm [48] and IFC. The vectors composed of the ADFs are used as the input of the K-means clustering algorithm. The number of keyframes to be extracted is determined by artificially setting the number of clusters. Then we compare the differences between the vectors representing frames and the vectors representing the cluster centers to obtain the final vectors representing the keyframe sequences.

The detailed process is: (i) to obtain keyframes. The most informative frames are extracted and the pose redundancy is removed, which can effectively compress and refine driver actions; (ii) to obtain keyframe sequences. The extracted keyframes are sorted according to the order of occurrence, which not only ensures that the extracted keyframe sequences have efficient spatiotemporal information but also reduces the number of ADF vectors sent to Module IV. Through this module, the accuracy of the model for action recognition can be improved.

**Step 1**: The K-means clustering algorithm is used to obtain keyframes. The basic principle of the algorithm is to group similar objects into the same cluster, and group dissimilar objects into different clusters. We take the value composition vector of the ADFs, that is, the vector angle feature value and the vector modulus ratio feature value, constructed in each frame as the input of the K-means clustering algorithm. We assume that the complete sequence of action is $\left\{x^{\left(1\right)}\right.$, $x^{\left(2\right)}$,⋯,$\left.x^{\left(N\right)}\right\}$, $x^{\left(i\right)}\epsilon R^{21}$, where N is the total number of frames in the action sequence, i is a frame in the sequence, $x^{\left(i\right)}$ is a 21-dimensional vector composed of the values of the 21 ADFs in the frame i, and $R^{\left(21\right)}$ is a collection of vectors composed of vectors in each frame in a complete sequence. In this paper, ADF-vectors representing frames are clustered into $K\left(K\leq N\right)$ clusters. The detailed process is as follows:

(1) Randomly select a K cluster centroid, and mark it as $u_{j}\varepsilon R^{N}$, j=1,2,⋯,K;
(2) Calculate the minimum D of the distance $D_{ij}$ from each sample $x^{\left(i\right)}$ to each centroid $u_{j}$, and classify the samples into the cluster j corresponding to the minimum distance, that is:

(7) $$D=arg\min\sum_{i=1}^{N}\sum_{j=1}^{K}∥x^{\left(i\right)}-u_{j}{∥}^{2}$$

(3) After the division, for each cluster j, recalculate the centroid:

(8) $$u_{j}=\frac{\sum_{i=1}^{N}r_{ij}x^{\left(i\right)}}{\sum_{i=1}^{N}r_{ij}}$$

where, $r_{ij}$ indicates whether the vector $x^{\left(i\right)}$ is classified into the cluster j, if it belongs to cluster j, then $r_{ij}=1$, otherwise $r_{ij}=0$.

(4) Repeat (2) and (3) until the cluster center remains unchanged, then the algorithm ends. Through the above process, K cluster centers which can be used as pre-selected keyframes are extracted, and each center is a 21-dimensional vector.

**Step 2**: Since the cluster center does not necessarily coincide with the ADF vectors completely, and does not have a time sequence, the IFC is used to further obtain the keyframe sequences. The detailed method is as follows:

(9) $$u_{j}=\left(\alpha_{1_{j}},\alpha_{2_{j}},\cdots ,\alpha_{13_{j}},\beta_{1_{j}},\beta_{2_{j}},\cdots ,\beta_{8_{j}}\right),j\in (1,2,\cdots ,K)$$

where, $u_{j}$ represents the vector of the cluster center of the cluster j, K is the number of keyframes to be extracted in an action sequence, $\alpha_{1_{j}}$, $\alpha_{2_{j}}$,…, $\alpha_{13_{j}}$ are the vector angle values, and $\beta_{1_{j}}$, $\beta_{2_{j}}$,…, $\beta_{8_{j}}$ are the modulus ratio values.

The ADF vector representing a frame in an action sequence is expressed as follows:

(10) $$x_{i}=\left(\theta_{1_{i}},\theta_{2_{i}},\cdots ,\theta_{13_{i}},r_{1_{i}},r_{2_{i}},\cdots ,r_{8_{i}}\right),i\in (1,2,\cdots ,N)$$

where, $x_{i}$ is the ADF vector of the frame i, N is the total number of frames in the action sequence, $\theta_{1_{i}}$, $\theta_{2_{i}}$,…, $\theta_{13_{i}}$ are the vector angle values, and $r_{1_{i}}$, $r_{2_{i}}$, …, $r_{8_{i}}$ are the modulus ratio values of the frame i.

By solving the minimum Euclidean distance C between the $u_{j}$ and $x_{i}$, we determine the correspondence between the cluster centers and the action sequence frames, which is expressed as follows:

(11) $$\begin{array}{cc} C & =Min(u_{j},x_{i})\\ & =\sqrt{{\left(\alpha_{1_{j}}-\theta_{1_{i}}\right)}^{2}+\cdots +{\left(\alpha_{13_{j}}-\theta_{13_{i}}\right)}^{2}+{\left(\beta_{1_{j}}-r_{1_{i}}\right)}^{2}+\cdots +{\left(\beta_{8_{j}}-r_{8_{i}}\right)}^{2}} \end{array}$$

In order to ensure that the extracted keyframes are consistent with the frames in the action sequences, it is necessary to mark the corresponding frame with the smallest distance as a keyframe, save the index of the frames, and finally sort by index to obtain the final keyframe sequence. Due to the small change of some actions, the ADF vectors of the frames are similar, which will cause the problem of inconsistency between the sequence of the extracted keyframes and the sequence in the video, but the recognition effect will not be affected.

### 4.4. Module IV

**Figure 7:**
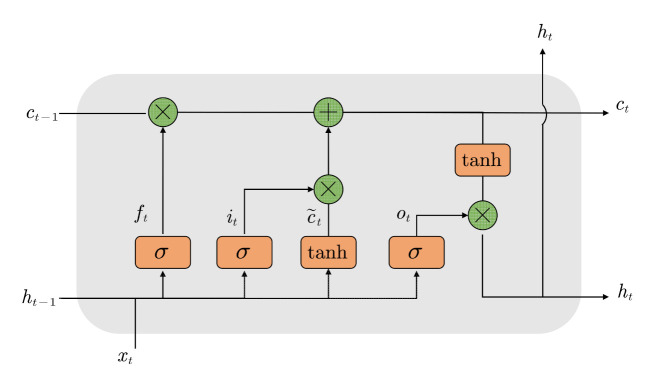
The basic structure of LSTM unit models.

An LSTM network is used to process the output results of Module III to extract the spatiotemporal features, and then transfer them to the softmax layer to output the action recognition results. The internal structure of the typical deep neural network with LSTM is a one-dimensional vector [49]. Figure 7 displays a basal LSTM neuron. Within LSTM models, there exist three gates to control and update the cell’s state: (1) inputs, (2) forget, and (3) output. The memory cell in each gate consists of a sigmoid neural net layer and a pointwise multiplication operation.

For time step t, the cell state can be updated by using the following equations:

(12) $$\left\{\begin{array}{l} i_{t}=\sigma \left(W_{xi}x_{t}+W_{hi}h_{t-1}+b_{i}\right)\\ f_{t}=\sigma \left(W_{xf}x_{t}+W_{hf}h_{t-1}+b_{f}\right)\\ o_{t}=\sigma \left(W_{xo}x_{t}+W_{ho}h_{t-1}+b_{o}\right)\\ {\tilde{c}}_{t}=\tanh\left(W_{xc}x_{t}+W_{hc}h_{t-1}+b_{c}\right)\\ c_{t}=f_{t}⊗c_{t-1}+i_{t}⊗{\tilde{c}}_{t}\\ h_{t}=o_{t}⊗\tanh\left(c_{t}\right) \end{array}\right.$$

where, σ stands for activate function sigmoid defined as $\sigma \left(x\right)={\left(1+e^{-x}\right)}^{-1}$, $i_{t}$, $f_{t}$, $o_{t}$ respectively stand for the outputs of the “input”, “forget”, and “output” gates. $c_{t}$ represents the long-term memory state of the cell at time t, ${\tilde{c}}_{t}$ denotes the candidate state value of $c_{t}$. $h_{t}$, and $x_{t}$ are the final output and initial input at time t. $W_{xi}$, $W_{hi}$, $W_{xf}$, $W_{xo}$, $W_{ho}$, $W_{xc}$, $W_{hc}$, $b_{i}$, $b_{f}$, $b_{o}$, and $b_{c}$ stand for the coefficient matrix and offset vector.

In the proposed model presented in Figure 2, a two-layer LSTM network is constructed to learn the ADF vectors of the keyframe sequence, so as to obtain the spatiotemporal features of the video sequences. In Figure 2, $\left\{f_{1}\right.$, $f_{2}$, ⋯, $\left.f_{n}\right\}$ are the ADF vectors that are constructed by Module II representing a keyframe sequence. Thus, from an input sequence $\left\{f_{1}\right.$, $f_{2}$, ⋯, $\left.f_{n}\right\}$, the memory cells in the two LSTM layers will produce a representation sequence $\left\{m_{1}\right.$, $m_{2}$, ⋯, $\left.m_{n}\right\}$. Finally, the feature vector $m_{n}$ at the last moment feeds into the softmax layer so that the driver distracted action can be identified.

## 5. Experiment

The hardware facility used in this study is a self-assembled desktop computer equipped with 3.2 GHz Intel i5-6500 CPU, 8 GB RAM, x-64 based processor, and NVIDIA GeForce GT1030 GPU, which sources from Beijing, China. All programs in this study were operated in Spyder 4.1.4. based on the Windows 10 operating system. The code was mainly implemented in Python language, and the network construction is based on the TensorFlow deep learning framework.

### 5.1. Selection of Hyper-Parameters

An important step in using the K-means clustering algorithm is to artificially determine the *K* value, which determines the number of keyframes to be extracted. Therefore, it is necessary to ensure that the extracted keyframes are not only representative but also to avoid data redundancy. Because the selection of hyper-parameters and the estimation of model performance need to be done on the same dataset, the traditional K-fold cross-validation is likely to cause an optimistic evaluation of model performance, because nested cross-validation techniques can overcome common problems related to overfitting and data bias when confined by limited data size. Moreover, it can also optimize hyper-parameters and provide an unbiased estimate of algorithmic generalization performance simultaneously [50]. So we chose the nested cross-validation technique instead of traditional K-fold cross-validation to optimize the parameters K and the parameters of LSTM in this research [50,51]. The nested cross-validation technique consists of inner cross-validation (CV) loops, which are used to optimize the hyper-parameters and the outer CV loop, which is applied to measure the generalization performance using the optimal hyper-parameter values of unseen test data.

**Figure 8:**
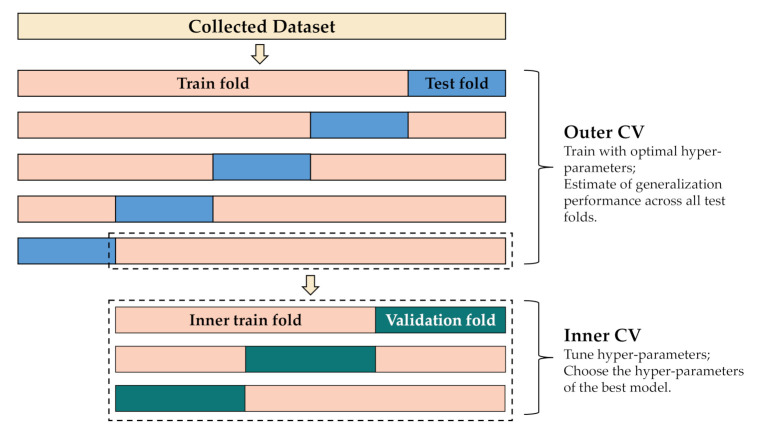
The flowchart of the fivefold nested CV.

The process for the nested cross-validation technique is shown in Figure 8. Our dataset is randomly split into five non-overlapping groups in the outer loop. In each group, two dissected subsets are called training sets and test sets respectively, and test sets are dedicated to model evaluation. In each iteration of the internal CV loop, the input training set is repeatedly split into validation and inner training sets using the threefold CV method. What is remarkable is that we divided samples of the dataset into five parts or three parts by action performers instead of randomly dividing. Table 5 shows the range of hyper-parameters. The selected values of three hyper-parameters to be optimized were combined to form various combinations. the inner training folds are used to derive different models by manually adjusting the hyper-parameters, while the validation set is used to estimate the performance of the model. The hyper-parameter corresponding to the highest classification accuracy of the inner CV loop is selected as the optimal hyper-parameter to train the external CV loop. 

The optimal hyper-parameter settings are shown in Table 6. We suspect that the selected keyframes are too few, resulting in the model’s acquired spatiotemporal information is not enough to perform similar actions. The selection of too many keyframes causes some non-keyframes to have a negative effect on recognition, that is, similar front-to-back correlations between different types of action sequences will interfere with recognition.

**Figure 9:**
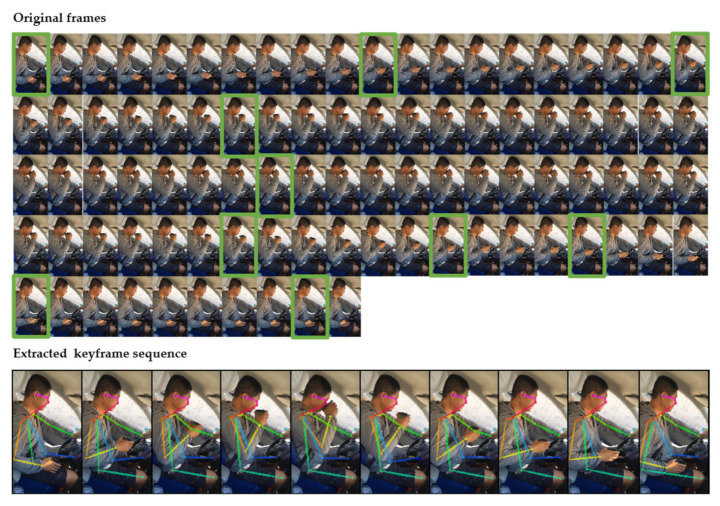
Qualitative results for our proposed keyframe sequences extraction method.

We qualitatively assessed whether the keyframe sequences we extracted can represent the complete action sequences. As shown in Figure 9, the distracting action completed by the performer of drinking water has a total of 90 original frames, and 10 keyframes are extracted, which have most of the information about the action. From this, we can conclude that the use of a few information frames from a portion of the action sequence is sufficient to accurately recognize the action.

### 5.2. Experimental Comparisons

In this section, we will evaluate the effectiveness of the proposed model from the following two perspectives.

(1) Comparison of different module combinations. We carry out different types of module combination experiments and perform a comparison with accuracy to reflect the effectiveness of our proposed Module II, Module III, and Module IV, respectively: (i) Combination of Module I and Module IV, including data processing, without feature construction and keyframe sequence selection. (ii) Combination of Module I, Module II and Module IV, and (iii) combination of Module I, Module III, and Module IV. It is noted that we apply Module II and Module III for separate experiments to prove the effectiveness of the fusion of these two modules for action recognition. (iv) The four-module framework proposed in this article. (v) Module IV was replaced with a support vector machine (SVM) algorithm. The SVM algorithm is used for comparison experiments, mainly because it is a machine learning algorithm that is often used in classification models and is between simple algorithms and neural network algorithms with excellent performance. Based on the structural risk minimization theory, SVM determines a hyperplane by finding several support vectors and divides the samples into two categories, so it was originally used to solve the binary classification problem. When faced with a multi-classification problem, it is indispensable to build a multi-classifier, among which common methods include the one-to-many method and one-to-one method. The parameter settings of the SVM used in the experiment are shown in Table 7. It is worth noting that the feature vectors representing keyframes are fed into the SVM separately.

What we need to do is to find the optimal parameters of the LSTM to get the most objective assessment of Module II and Module III’s performance, rather than controlling these parameters to be consistent across the four models. Therefore, on the premise that the LSTM network is used to extract temporal information, the parameter settings of the LSTM network in the four comparative experiments (experiment (i)–(iv)) are inconsistent, as shown in Table 8.

Table 9 summarizes the detailed data and results of the comparison of different module combinations. It can be seen from the experimental results that Module II and Module III increase the accuracy rate from 82% to 90.25% and 86.75% respectively, and the combination of the two modules increases the accuracy rate by 92.13%. By using the combination of Module II and Module III, instead of using only one of the feature processing methods, it is very intuitive to greatly increase the robustness of the detection. In addition, the proposed method of feature processing can well eliminate the impact of individual differences of different drivers and experimental distance changes on the recognition accuracy, and the improvement of accuracy also proves the correctness and effectiveness of the method. Through the comparison of experiment (iv) and experiment (v), using LSTM is better than SVM. This result proves that spatiotemporal features contain more information expression than single spatial features, which is conducive to action recognition.

(2) Comparison with the state-of-the-art methods. In the field of DAR, most studies still focus on improvements of the CNN model, which means we cannot fairly compare our approach with them. We set up five comparative experiments with traditional CNN-LSTM. In the experiments, we apply transfer learning [52] to save computing resources and avoid local optimization problems. Transfer learning requires a pre-trained network that has been trained for some tasks. In transfer learning, we do not need to modify the hidden weights of the pre-trained network, which are just used to extract the features of the new task. A typical transfer learning technique is to append a new fully connected network to the end of the pre-trained network. In the literature [18,21,53,54], fine-tuned VGG16 and Inception V3 have been successfully applied to solve various problems. Therefore, we selected these two pre-trained CNN models which were trained for image classification in a subset of ImageNet. This subset, published in the ImageNet Large-Scale Visual Recognition Challenge, consists of approximately 1000 different categories and 1.2 million images. We use the Keras implementations of Inception V3 and VGG16 models for transfer learning, which consists of 11 blocks/311 layers and 5 blocks/19 layers, respectively. The model details are shown in Table 10. In addition to these blocks, there is a final block that divides the training set into 1000 classes at the end of each model by the fully connected and softmax layers. Because driver actions are divided into eight classes in this study, much smaller than those of the pre-trained model, it is necessary to fine-tune the network to fit our dataset. Therefore, the last block of the pre-trained network is replaced by the final block we designed ourselves, as shown in Table 11.

After determining the structure of the CNN network, we combined the fine-tuned CNN with the self-made LSTM forming two CNN-LSTM network models. The CNN models are applied to each frame of the video sequences to obtain the class probability estimates, which are fed into LSTM as image spatial information. The structure of LSTM is shown in Table 12. Two different models were eventually identified, which were CNN-LSTM models.

After determining the model, the self-made dataset was used for training. Firstly, the self-made dataset was divided into 80% training and 20% testing, and then the training set was divided into 70% training and 30% validation, which is used for hyper-parameter optimization. In this process, the Adam optimizer [55], which aims to learn the problem faster by adapting or optimizing the learning rate, was used for model training for 40 epochs. The results of the experiments are shown in Table 13. It can be seen that the model we proposed is more competitive by comparing the accuracy of VGG16-LSTM and Inception V3-LSTM. The results also prove that handcrafted features derived from OpenPose are more representative than automatically generated features from CNN.

**Figure 10:**
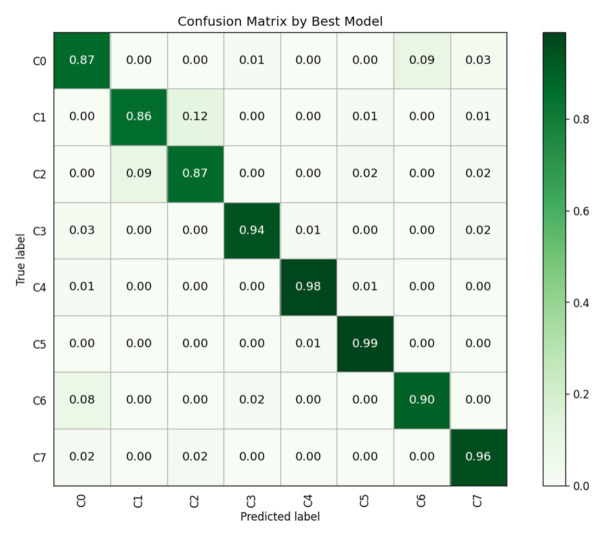
Confusion matrix by our proposed model.

The average recognition accuracy of the model we proposed was 92.13%, which can meet the needs of practical applications. In order to fully verify the classification effect of the framework, we show the confusion matrix of the best model in Figure 10. As can be seen from the figure, there is still some confusion about similar actions, such as eating and drinking, driving safely, and talking to passengers, the reason being that these two actions are too similar. However, for the most common and more traffic accident-prone movements such as making phone calls, texting, and makeup [56], the model we propose achieves high-precision recognition. Besides, misidentifying eating as drinking and vice versa has little effect on our model’s warnings about distractions. In the future, better feature representation will be needed to distinguish between the misidentification of safe driving and talking to passengers. In addition, the processing time of our proposed method is 3.48 s when the server GPU acceleration is strong enough. In real life, distractions such as making phone calls and texting tend to last more than 10 s, and the real cause of traffic accidents is usually a long period of continuous action [9]. Compared with the duration of these actions, the processing time of the model we proposed is negligible to meet the needs of practical application.

## 6. Conclusions and Feature Works

This paper proposes a method of driver distraction recognition based on RGB video, which emphasizes the importance of temporal features and fills the gap in the DAR field. The hybrid deep learning model we proposed does not only rely on spatial features but also extracts efficient spatiotemporal features from the driver’s action sequence to improve the accuracy and robustness of the driver’s distracted action recognition. The realization of this framework mainly relies on three methods (i) computer vision method, namely CNN-LSTM architecture is used as the basic framework, (ii) feature construction based on joint points, and (iii) keyframe sequence extraction. The improved feature construction method we proposed can weaken individual differences and improve the generalization ability of the model when the relative distance between the driver and the camera changes, or there are differences in height, weight, and body proportions between different drivers. The extracted keyframes enhance the process by providing information that is free from redundancy but carries the most relevant details about the action that exists in the motion. Finally, for thorough and detailed performance evaluations of every module and the model, we designed two sets of comparative experiments. The first group of experiments has compared the influence of different module combinations on distracted action recognition. The results show that the model we proposed has higher recognition accuracy than other module combinations. In this group of experiments, the comparisons of Module II and Module III for separate experiments and the combination of these two modules prove the effectiveness of the modules for action recognition. The second group of experiments was designed to compare our methods with the state-of-the-art methods. We have conducted sufficient experiments based on a custom dataset and our proposed method comparatively produced very competitive results.

Due to the constraint effect of the dataset itself on the neural network, the increase in the number of datasets is helpful for the feature extraction and generalization capabilities of the model. Although a high-quality video dataset was collected, a more diverse dataset is required to cover more scenarios and more kinds of drivers (e.g., different races and body shapes), which is the focus of future work. On the technical side, in the future, our approach can be applied from the following two aspects:

- Complement the method we proposed with existing methods for detecting driver who is fatigued, drunk, or sleeping to achieve a complete driver distraction monitoring system.
- The approach proposed in this paper can be combined with other intelligent methods, such as the trajectory prediction method [57] and interpretable decision-making method [58], to build a complete intelligent system to support autonomous vehicles, which can assist in detecting whether the driver can take over vehicle control under emergencies.

## Figures and Tables

**Table 1:** Comparison of mainstream architectures in the field of HAR.

Architecture	Introduction	Precision	Speed
Two-stream [11]	The dense optical flow is calculated for every two frames in the video sequence. Then the CNN model is trained on image and dense optical flow respectively.	high	slow
3D-ConvNet [12]	Temporal and spatial features of the video sequence are extracted by a 3D convolution kernel. The motion of the video stream can therefore be captured.	low	fast
CNN-LSTM [13]	The CNN-LSTM architecture can be understood as the series structure in the circuit, which can extract spatial and temporal information from a video sequence.	high	fast

**Table 2:** Actions of the drivers.

NO	Action
C0	Safe driving
C1	Eating while driving
C2	Drinking while driving
C3	Manipulating dashboard controls
C4	Texting with the right hand
C5	Taking on a phone with the right hand
C6	Talking with passengers
C7	Doing makeup while driving

**Table 3:** Eighteen joint points of the human body.

No.	Joint Point	NO.	Joint Point
0	Nose	9	Knee Right
1	Shoulder Center	10	Ankle Right
2	Shoulder Right	11	Hip Left
3	Elbow Right	12	Knee Left
4	Wrist Right	13	Ankle Left
5	Shoulder Left	14	Eye Right
6	Elbow Left	15	Eye Left
7	Wrist Left	16	Ear Right
8	Hip Right	17	Ear Left

**Table 4:** Vector module ratio.

Abbreviation	Modulus Ratio	Abbreviation	Modulus Ratio
$r_{1}$	$r_{O,2}$	$r_{5}$	$r_{O,6}$
$r_{2}$	$r_{O,3}$	$r_{6}$	$r_{O,7}$
$r_{3}$	$r_{O,4}$	$r_{7}$	$r_{4,0}$
$r_{4}$	$r_{O,5}$	$r_{8}$	$r_{7,0}$

**Table 5:** The range of values for the hyper-parameters.

Hyper-Parameter	Value
K	6/8/10/12/14
Hidden layer Units	32/64/128
Learning Rate	0.01/0.001/0.0001

**Table 6:** The optimal hyper-parameter settings.

Module III and IV	Parameter	Value
K-means clustering algorithm	K	10
LSTM networks	Number of Hidden Layers	2
	Hidden layer Units	32/64
	Batch Size	10
	Epochs	100
	Learning Rate	0.001
	Optimizer	Adam
	Loss Function	Sparse_categorical_crossentropy
	Validation Frequency	1

**Table 7:** Parameter setting of the SVM.

Kernel Function	Construction Method of Multi-Class Classifier	Number of SVM
Gauss kernel	One to one	28

**Table 8:** Parameter of LSTM networks in experiments (i)–(iv).

Experiment	Hidden Layer Units	LSTM Layers Params	Total Params
(i)	64/128	25,856/98,816	125,704
(ii)	32/64	6912/24,832	32,264
(iii)	64/128	25,856/98,816	125,704
(iv)	32/64	6912/24,832	32,264

**Table 9:** Comparison of methods on self-made datasets. The results are presented in the order of experiments (i–v), and the result of using the completed architecture we proposed in Figure 2 is shown in bold text. Note: joint points (JP), module ratio (MR), vector angle (VA), keyframe sequences (KFS), keyframes (KF). For more details, see Section 4.2 and Section 4.3.

Experiment	Approach	Features	Accuracy
(i)	Module I+ IV	JP	82%
(ii)	Module I+ II+ IV	MR+ VA	90.25%
(iii)	Module I+ III+ IV	JP+ KFS	86.75%
(iv)	Module I+ II+ III+ IV	MR+ VA+ KFS	92.13%
(v)	(Module I+ II+ III)+ SVM	MR+ VA+ KF	77.02%

**Table 10:** VGG16 and Inception V3 networks.

CNN Model	Block(s)	Layer(s)
VGG16	0	0
	1	4
	2	7
	3	11
	4	15
	5	19
Inception V3	0	0
	1	41
	2	64
	3	87
	4	101
	5	133
	6	165
	7	197
	8	229
	9	249
	10	280
	11	311

**Table 11:** The final block.

Block	Layer(s)
Final block	Flatten
	Full connected-1024 with ReLU
	Batch normalization
	Dropout (0.5)
	Fully connected-8 with softmax

**Table 12:** LSTM model constructed for CNN-LSTM.

LSTM Models Layers
Input (90 frames × 8 Class probabilities)
LSTM-32
Dropout (0.3)
Dense-8 with softmax

**Table 13:** Comparison of methods on the custom dataset.

CNN Model	Operation	Features	LSTM Model	Accuracy
VGG16	Transfer learning	Deep neural features	√	75.94%
Inception V3			√	79.58%
OpenPose	Construct ADFs	Handcrafted features based on deep neural features	√	92.13%

## Data Availability

The data presented in this study are available on request from the corresponding author. The data are not publicly available as they involve the subsequent application of patent, software copyright and the publication of project deliverables.
